# C-Type Natriuretic Peptide (CNP) Inhibition of Interferon-γ-Mediated Gene Expression in Human Endothelial Cells In Vitro

**DOI:** 10.3390/bios8030086

**Published:** 2018-09-14

**Authors:** Amy Day, Zoe Jameson, Carolyn Hyde, Bigboy Simbi, Robert Fowkes, Charlotte Lawson

**Affiliations:** 1 Cardiovascular and Inflammation Biology Group, Comparative Biomedical Sciences, Royal Veterinary College, Royal College Street London, NW1 0TU, UK; Amy.Day@gstt.nhs.uk (A.D.); zjameson3@rvc.ac.uk (Z.J.); 2 Endocrine Signalling Group, Royal Veterinary College, University of London, Royal College Street, London NW1 0TU, UK; bsimbi@rvc.ac.uk; 3 Bio-Analysis Centre, London Bioscience Innovation Centre, Royal College Street, London NW1 0NH, UK; cali@b-ac.co.uk

**Keywords:** C-type natriuretic peptide, endothelium, inflammation, cardiovascular, interferon gamma, indoleamine-2,3-dioxygenase

## Abstract

Cardiovascular diseases, including atherosclerosis, now account for more deaths in the Western world than from any other cause. Atherosclerosis has a chronic inflammatory component involving Th1 pro-inflammatory cytokines such as IFN-γ, which is known to induce endothelial cell inflammatory responses. On the other hand CNP, which acts via its receptors to elevate intracellular cGMP, is produced by endothelium and endocardium and is upregulated in atherosclerosis. It is believed to be protective, however its role in vascular inflammation is not well understood. The aim of this study was to investigate the effects of CNP on human endothelial cell inflammatory responses following IFN-γ stimulation. Human umbilical vein endothelial cells were treated with either IFN-γ (10 ng/mL) or CNP (100 nm), or both in combination, followed by analysis by flow cytometry for expression of MHC class I and ICAM-1. IFN-γ significantly increased expression of both molecules, which was significantly inhibited by CNP or the cGMP donor 8-Bromoguanosine 3’,5’-cyclic monophosphate (1 µm). CNP also reduced IFN-γ mediated kynurenine generation by the IFN-γ regulated enzyme indoleamine-2,3-deoxygenase (IDO). We conclude that CNP downmodulates IFN-γ induced pro-inflammatory gene expression in human endothelial cells via a cGMP-mediated pathway. Thus, CNP may have a protective role in vascular inflammation and novel therapeutic strategies for CVD based on upregulation of endothelial CNP expression could reduce chronic EC inflammation.

## 1. Introduction

According to the latest statistics published by the American Heart Association cardiovascular disease (CVD) is the largest cause of death worldwide and accounts for over 750,000 deaths in the USA annually [1,2]. The importance of the endothelium is well established for the initial development and subsequent advancement of CVD, having a large influence on maintenance of blood vessel tone via production of nitric oxide and other vasoactive factors. Furthermore, a healthy endothelium is responsible for the maintenance of an anti-thrombotic environment via the production of tissue factor pathway inhibitors and thrombomodulin [3,4]. There is now compelling evidence that chronic systemic inflammation also has a major impact on progression of CVD, with accelerated secondary CVD being noted in obese individuals, and as a risk factor for patients with diabetes or autoimmunity [1]. 

There is very strong evidence that CD4^+^ T helper type 1 (Th1) cells are present in early atheromatous lesions and contribute to lesion progression [5], including production of the pro-inflammatory cytokine, interferon gamma (IFN-γ). IFN-γ has many pro-inflammatory effects on vascular endothelial cells (EC), most notably enhancing the expression of adhesion molecules involved in firm adhesion of both monocytes (VCAM-1), and other leukocyte populations (ICAM-1 [6]). It also increases expression of MHC class I and MHC Class II in human EC [7], which may lead to further activation of antigen specific CD8 and CD4^+^ T cells, thus contributing to plaque progression.

IFN-γ also has a number of immuno-regulatory functions, one of the most well described is its upregulation of indoleamine-2,3-deoxygenase (IDO), an inducible enzyme that reduces tryptophan availability by catalyzing its breakdown to kynurenine for reviews see [8,9,10,11]. This reduction in bioavailable tryptophan in the local microenvironment of the inflamed vasculature could potentially reduce the activity of highly metabolic cells such as T lymphocytes and myeloid cells and therefore reduce the chronic immune response. 

Atrial- and B-Type natriuretic peptides (ANP and BNP, respectively) are well characterised hormones that exert profound effects on the cardiovascular system, as well as having established anti-inflammatory roles in the endothelium and other tissues [12,13,14,15]. C-type natriuretic peptide (CNP) is a 22 amino acid peptide, the third member of the natriuretic peptide family, identified first in extracts from porcine brain [16]. It is known to have actions on central regulation of vasoactive hormones such as vasopressin and adrenocorticotrophin hormone release, but has also been shown to have direct vasodilatory effects, in particular on smooth muscle relaxation. Coupled with the finding that it is less well expressed in heart than other family members and its abundance in endothelium, it has been hypothesized to be a third, so called, endothelial derived hyperpolarizing factor (EDHF), alongside nitric oxide and prostacyclin [17,18]. CNP has also been described to have broadly “anti-inflammatory” effects on endothelial cells in vitro [19,20,21], to be anti-fibrotic [22,23,24] and to be cardioprotective [25,26,27,28].

We hypothesized that as CNP has been shown to be abundantly expressed in endothelial cells and to have broadly anti-inflammatory effects on EC it can act as a protective brake, specifically acting on pro-inflammatory molecule expression to limit leukocyte emigration and IDO expression to limit subsequent activation after an inflammatory insult. We measured the effect of CNP administration on endothelial pro-inflammatory gene expression including ICAM-1, MHCI, MHCII and IDO activity after treatment of human umbilical vein endothelial cells with IFN-γ.

## 2. Materials and Methods

### 2.1. Materials

All reagents were from Sigma (Poole, UK) unless otherwise stated. All tissue culture reagents were from Sigma or Gibco (ThermoFisher, Loughborough, UK), and plastics were from Nunc (ThermoFisher; Loughborough, UK). 

### 2.2. Cell Culture

Umbilical cords were collected from The Royal London Hospital with approval from the East London Research Ethics Committee and according to the Declaration of Helsinki. Human umbilical vein endothelial cells (HUVEC) were isolated according to a modified method of Jaffe [29] and maintained as described previously [30]. Cells were seeded onto plates pre-coated with gelatin at 2 × 10^5^ cells/well (6 well) or 5 × 10^4^ cells/well (24 well) plates as appropriate. They were allowed to adhere overnight before treatment with IFN-γ 10 ng/mL (Insight Biotech, Wembley, UK), 100 nm CNP (Sigma, Poole, UK) for up to 72 h, 1 µm 8-bromo-cGMP (Sigma, Poole, UK) or 100 µm 8-(4-Chlorophenylthio)-guanosine 3’,5’-cyclic monophosphate (8-CPT-cGMP; Sigma, Poole, UK), for 48 h. 

### 2.3. Flow Cytometry

HUVEC were treated on 24 well plates for up to 72 h as above. The supernatants were removed and snap frozen at −80 °C for further analysis. Cells were harvested by trypsinization. Cells were centrifuged and the pellet resuspended in 2 mL PBS divided between four 5 mL FACS tubes and re-centrifuged. Pellets were resuspended in 50 μL PBS and primary antibodies added at predetermined concentrations (anti-human ICAM-1 clone 6.5B5 (a gift from Professor DO Haskard, Imperial College UK); anti-MHC class I clone W6/32 (ATCC); MHC class II clone L243 (ATCC)). Cells were held on ice for 30 min, washed with 2 mL ice cold PBS, centrifuged, pellets were resuspended in 50 μL cold PBS with predetermined concentration of FITC-conjugated Goat anti-mouse-Ig antibody (Jackson; Stratech Luton, UK) and incubated on ice for 30 min followed by a wash with 2 mL ice cold PBS, and centrifugation. Pellets were resuspended in 0.5 mL PBS/0.5% formaldehyde and held at 4 °C before analysis by flow cytometry using a FACS CANTO II with FACS DIVA software (BD Biosciences; Oxford, UK). The flow cytometer was calibrated daily with CS&T beads (BD Biosciences) according to the manufacturer’s instructions. Cell populations were gated on FSC/SSC and using a negative control of FITC-conjugated secondary antibody alone. 

### 2.4. cGMP Assay

Cells were treated with CNP in quadruplicate in 24 well plates for 30 min at 37 °C before removal of culture medium and lysis and fixation with 750 μL 100% Ethanol for 5 min at RT before freezing at −20 °C until analysis using cGMP EIA (R&D, Oxford, UK) according to the manufacturer’s instructions.

### 2.5. Mass Spectrometry for Tryptophan Metabolites

Four metabolites of tryptophan were prepared at 1 mg/mL for use as standards Kynurenine (K), 2,3-Pyridinecarboxylic acid (PCA), 3 Hydroxy DL Kynerenine (HDLK) and 3 Hydroxyanthranilic acid (HAA) (All from Sigma). K, PCA and HAA were dissolved in 500 µL of water and 500 µL methanol. HDLK was dissolved in 600 µL methanol, 400 µL water and 1 µL formic acid. Standards were; 10 µg/mL, 100 ng/mL and 10 ng/mL.

Samples were thawed at room temperature and 10 µL of each sample was diluted with 495 µL of water and 495 µL of methanol.

A Shimadzu LCMS8040 was used—a triple quadruple mass spectrometer with high sensitivity, high speed and high reliability. Analysis of both positive and negative ions is possible in the same experiment due to the ultrafast polarity switching capabilities. LabSolutions software was used for the acquisition and analysis of data. A Phenomenex (Macclesfield, UK) Kinetex PFP (50 × 2.1 mm i.d., 2.6 µm, 100 Å) with mobile phases A (0.1% formic acid in water, *v/v*) and B (0.1% formic acid in Methanol, *v/v*) was used. The column was kept at 40 °C. All analytes were detected in positive ion multiple reaction monitoring (MRM) mode. The flow rate, at all times, was 0.25 mL/min. The column effluent was delivered to the mass spectrometer with no split. If not otherwise noted, an injection volume of 1 μL was used. All MS parameters were optimized by the auto optimization program. The ESI source was operated under standard conditions of Nebulising Gas at 3 L/min, DL temperature at 250 °C, a heat block temperature at 400 °C and drying gas flow at 15 L/min.

### 2.6. Data Analysis 

Post-acquisition analysis of flow cytometry data was using FACS DIVA II software (BD Biosciences, Oxford, UK), FloJo v10 (FLoJo LLC, Ashland, OR, USA) and Flowing Software v2.5.1 (University of Turku, Finland) and data is presented as fold increase in median fluorescence intensity over untreated cells; mean ± SEM. All statistical analyses were performed using Prism 7 (GraphPad Software Inc., CA, USA). One-way ANOVA followed by Bonferroni post-test or Independent T tests were used as appropriate, with *p* < 0.05 (*) considered as statistically significant. Mann–Whitney tests were used to analyze data that were not normally distributed. All experiments were performed on at least three separate isolates of HUVEC and data are presented as mean ± SEM. 

## 3. Results

### 3.1. CNP Reduces IFN-γ Mediated Expression of Pro-Inflammatory Molecules on the Surface of HUVEC.

**Figure 1:**
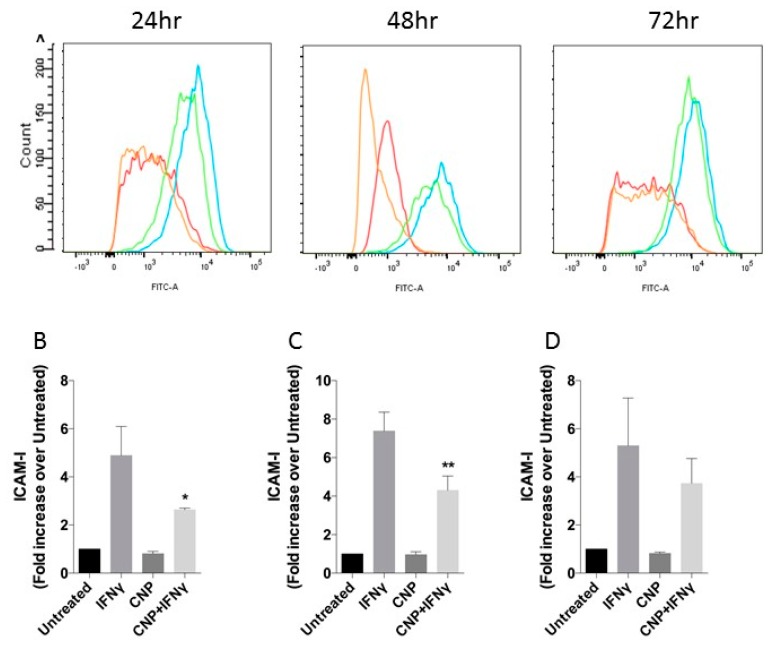
ICAM-1 expression in HUVEC after incubation with IFN-γ alone or in combinations with CNP for up to 72 h. (**A**) Representative flow cytometry histograms for ICAM-1 after incubation of HUVEC alone (**Red**) or with IFN-γ (**Blue**), CNP (**Orange**) or IFN-γ and CNP (**Green**) for the indicated times. (**B**–**D**) Mean Fluorescence Intensity (expressed as fold increase over MFI of untreated cells, which range from 820 to 3089; mean ± SEM) for ICAM-1 on untreated HUVEC or after 24 h (**B**), 48 h (**C**) or 72 h (**D**) treatment with IFN-γ alone or in combination with CNP; n = 3 HUVEC isolates (* *p* < 0.05, ** *p* < 0.01, significantly different from IFN-γ alone).

**Figure 2:**
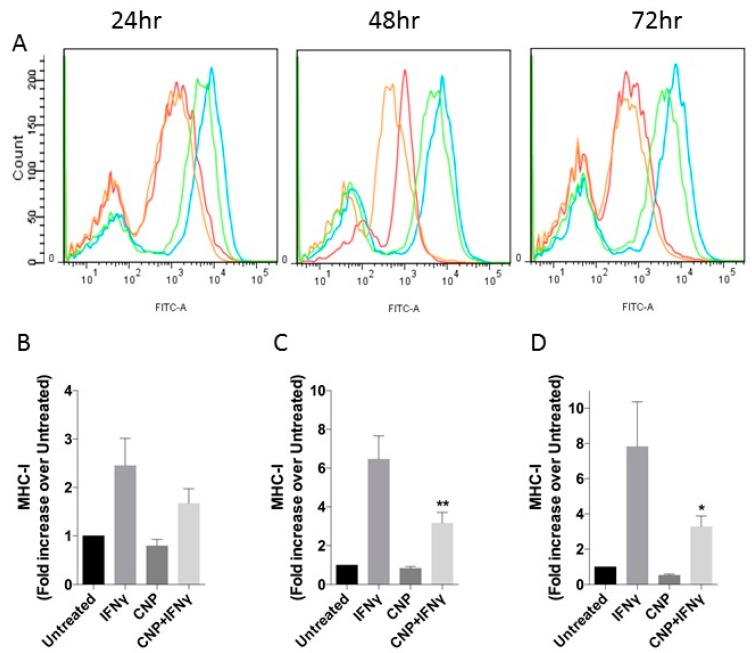
MHC class I expression in HUVEC after incubation with IFN-γ alone or in combinations with CNP for up to 72 h. (**A**) Representative flow cytometry histograms for MHC-I after incubation of HUVEC alone (**Red**) or with IFN-γ (**Blue**), CNP (**Orange**) or IFN-γ and CNP (**Green**) for the indicated times. (**B**–**D**) Mean Fluorescence Intensity (expressed as fold increase over MFI of untreated cells, which ranged from 2079 to 6549; mean ± SEM) for MHC-I on untreated HUVEC or after 24 h (**B**), 48 h (**C**) or 72 h (**D**) treatment with IFN-γ alone or in combination with CNP; *n* = 3 HUVEC isolates (* *p* < 0.05, ** *p* < 0.01, significantly different from IFN-γ alone).

**Figure 3:**
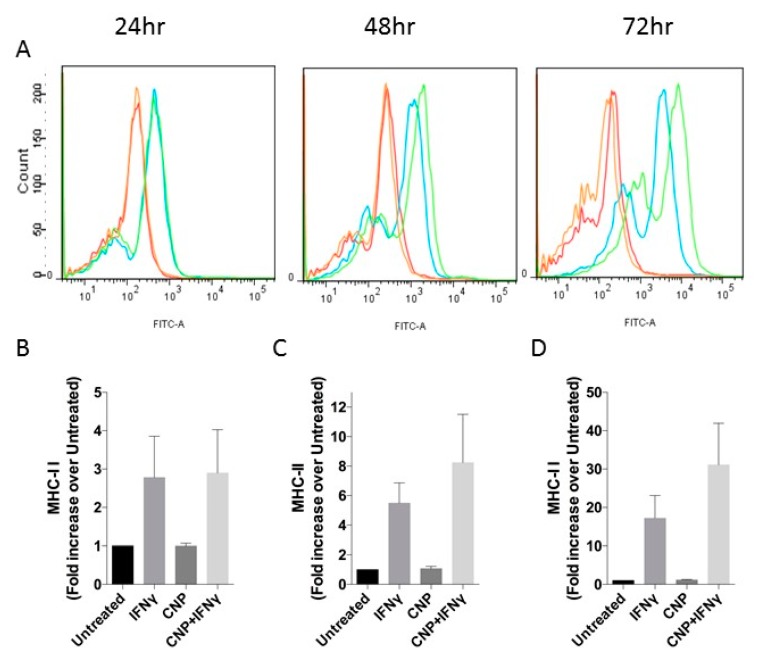
MHC class II expression in HUVEC after incubation with IFN-γ alone or in combinations with CNP for up to 72 h. (**A**) Representative flow cytometry histograms for MHC-II after incubation of HUVEC alone (**Red**) or with IFN-γ (**Blue**), CNP (**Orange**) or IFN-γ and CNP (**Green**) for the indicated times. (**B**–**D**) Mean Fluorescence Intensity (expressed as fold increase over MFI of untreated cells, which ranged from 187 to 216; mean ± SEM) for MHC-II on untreated HUVEC or after 24 h (**B**), 48 h (**C**) or 72 h (**D**) treatment with IFN-γ alone or in combination with CNP; *n* = 3 HUVEC isolates.

As shown in Figure 1, and as has been described previously in the literature [31,32] IFN-γ induced 4.9 ± 1.2-fold increase in ICAM-1 expression above basal levels in HUVEC after 24 h treatment, increasing to 7.4 ± 0.9-fold after 48 h and maintained for at least 72 h. Alone, CNP had no effect on ICAM-1 cell surface expression; however, it caused a significant reduction in the IFN-γ mediated response at both 24 h (to 2.6 ± 0.06-fold, *p* < 0.05) and 48 h (to 4.3 ± 0.7-fold, *p* < 0.05) with the trend continuing out to 72 h treatment (3.7 ± 1.0-fold, *p* = 0.24). Similarly, CNP did not affect basal MHC class I expression when administered alone to HUVEC for up to 72 h (Figure 2) but significantly reduced IFN-γ mediated increased surface expression after 48h co-treatment (6.4 ± 1.2-fold vs. 3.2 ± 0.6-fold, *p* < 0.05) and 72 h (7.8 ± 2.5-fold vs. 3.3 ± 0.6-fold *p* < 0.05). In contrast (Figure 3), although IFN-γ increased the expression of MHC class II on the surface of HUVEC at each time-point (2.8 ± 1.1-fold, 5.5 ± 1.4-fold, 17.2 ± 6.0-fold, at 24 h, 48 h and 72 h, respectively), CNP failed to significantly alter these responses.

### 3.2. CNP Induces cGMP Release in HUVEC

**Figure 4:**
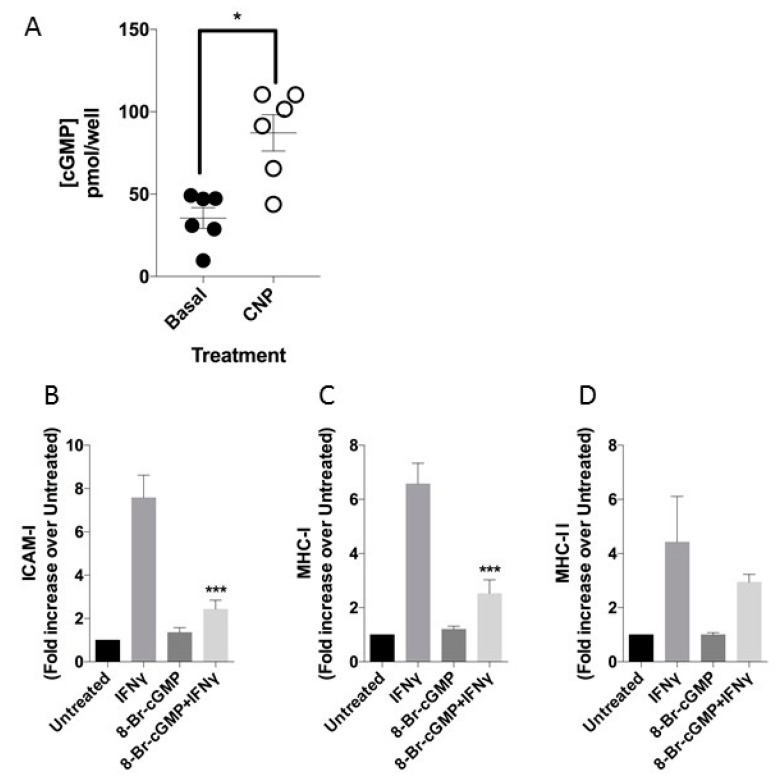
cGMP mediated effects on cell surface receptor expression in HUVEC. (**A**) CNP-stimulated cGMP accumulation in HUVEC isolates, after 30 min stimulation with 100 nm CNP in the presence of 1 mm IBMX. Data shown are means ± SEM pooled from 6 independent isolates (*n* = 6), each performed in duplicate (* *p* = 0.015, significantly different from Basal). (**B**–**D**) Effect of 8-bromo-cGMP on IFN-γ mediated ICAM-1 (**B**), MHC-I (**C**), and MHC-II (**D**) expressed as fold increase over MFI of untreated cells expression after 48hr treatment (which ranged from 572 to 819, 1313 to 2012, 176 to 230, for ICAM-1, MHC-I and MHC-II, respectively). *n* = 3 HUVEC isolates (* *p* < 0.05, *** *p* < 0.001, significantly different from IFN-γ alone).

CNP exerts the vast majority of its effects via the guanylyl cyclase B (GC-B) receptor and cGMP generation [33,34]. We therefore measured cGMP accumulation in HUVEC stimulated with 0 or 100 nm CNP in the presence of 1 mm IBMX. As shown (Figure 4A), CNP stimulated total cGMP accumulation in HUVEC isolates, to 2.5 ± 0.3-fold above basal (*p = 0.015). To determine whether the observed effects of CNP on IFN-γ-induced ICAM-1 and MHC class I expression in HUVEC were mediated via cGMP, we used a cell permeable cGMP agonist (8-Br-cGMP) to mimic the observed cGMP increase in response to CNP. As shown (Figure 4B,C), 1 mm 8-Br-cGMP caused an identical inhibition of the IFN-γ-mediated increases in ICAM-1 (7.6 ± 1.04-fold vs. 2.4 ± 0.4-fold, *p* < 0.001) and MHC class I expression (6.6 ± 0.8-fold vs. 2.5 ± 0.5-fold, *p* < 0.001). However, similar to CNP, 8-Br-cGMP failed to alter the effects of IFN-γ on MHC class II expression (Figure 4D). 8-CPT-cGMP also reduced IFN-γ mediated increase in MHC class I (Supplementary Figure S1). Collectively, these data support a role for GC-B/cGMP signaling in attenuating the effects of IFN-γ in HUVEC.

### 3.3. CNP Inhibits IFN-γ Induced Upregulation of Tryptophan Metabolism 

**Figure 5:**
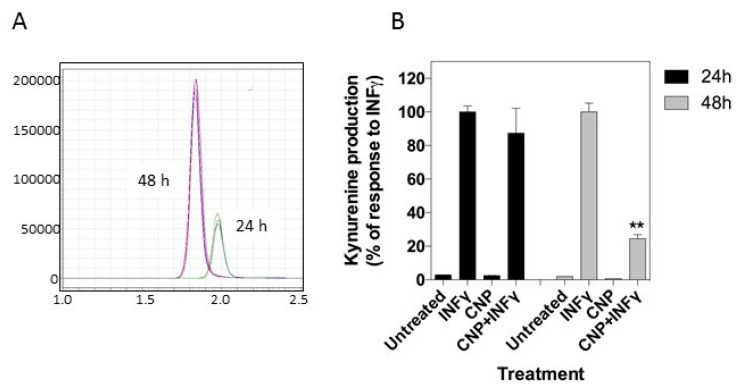
CNP downregulates IFN-γ-mediated tryptophan metabolism in HUVEC. (**A**) Representative chromatogram of kynurenine production following IFN-γ treatment. (**B**) Kynurenine production from untreated HUVEC or after 24 h and 48 h treatment with IFN-γ alone or in combination with CNP. Data shown are representative from a single HUVEC isolate, expressed as % IFN-γ -stimulated kyunurenine production), and performed in duplicate. (** *p* < 0.01, significantly different from IFN-γ alone).

IFN-γ has previously been shown to upregulate expression of indolamine 2,3-dioxygenase (IDO), an inducible enzyme found at sites of immune privilege and thought to be important for exerting the anti-microbial effects of IFN-γ [11]. The enzyme works to reduce tryptophan availability, catalyzing the first step of the pathway to kynurenine, which can be measured by mass spectrometry in vivo in plasma or in vitro in cell culture medium. As shown in Figure 5, IFN-γ stimulation induced a significant increase in kynurenine in the culture supernatant and this was significantly reduced by co-treatment with CNP after 48 h (100.0 ± 5.3% vs. 24.5 ± 2.4%, *p* < 0.01). This suggests that as well as potentially downregulating leukocyte adhesion and trans-endothelial migration during endothelial inflammation it may have an opposing effect of maintaining function of those leukocytes which are able to adhere and migrate at inflammatory sites in the vasculature. 

## 4. Discussion

Here, we have shown that CNP, a natriuretic peptide shown to be broadly cardioprotective [35] and known to be highly expressed by the endothelium [36] is able to downregulate IFN-γ-mediated gene expression in human endothelial cells in vitro. In particular, we have shown that CNP significantly inhibited the IFN-γ increase in ICAM-1 expression on the cell surface of HUVEC. ICAM-1 is an adhesion molecule involved in firm adhesion and trans-endothelial migration of leukocytes including neutrophils, monocytes and both T and B cells, and is also important for immunological synapse formation during T cell activation (for review see [37]), so by downregulating expression to basal levels CNP may prevent leukocyte accumulation during vascular inflammation, that could lead to the early stages of fatty streak formation. Likewise, by reducing ICAM-1 expression on the endothelium, immunological synapse formation will be compromised which could limit T cell accumulation. Thus, development of the pro-inflammatory environment leading to lesion formation will be reduced by some degree. ICAM-1 is a ligand for β-2 integrin family members, including αx/β2 (CD11c/CD18), the α-subunit (CD11c) having recently been shown to be upregulated in IDO/*ApoE*^−/−^ mice [38]. However, VCAM-1 has been shown to be an alternative ligand for CD11c [39], which Polyzos et al. [40] recently demonstrated was upregulated in *ApoE*^−/−^ mice and aortic endothelial cells treated chronically with 1-MT to inhibit IDO. Polyzos et al. [40] also showed that CCL2 was increased. Taken together these could explain the increased macrophage accumulation after IDO blockade in these two animal models. Interestingly, CNP has previously been shown to downregulate secretion of CCL2 from THP-1 human macrophage cell line in vitro [41] and there are differences in expression patterns of VCAM-1 in murine and human endothelial cells [7,42]. Future work should examine the effect of CNP on other IFNγ-mediated responses including expression of VCAM-1 and CCL2, or the effects of CNP on leukocyte adhesion and trans-endothelial migration.

We have also demonstrated that CNP reduced IFN-γ-mediated increases in MHC I expression. This could additionally contribute to a brake on CD8^+^ T cell immune responses in the inflamed environment. Cytotoxic T cells have been identified in advanced atherosclerotic lesions and may contribute up to 50% of the lymphocyte population [43]. Activated CD8^+^ T cells have been shown to efficiently migrate into the intima of both healthy and diseased arteries in vitro [43]. Interestingly, we did not see an effect of CNP on IFN-γ induced MHC class II expression, suggesting that CNP exerts a selective inhibition of CD8 T cell/NK cell accumulation, whilst allowing CD4^+^ T cell activity. There is evidence that certain subsets of CD4^+^ T cells (Th2-like, Treg) may be beneficial in reducing atherosclerotic lesion formation [3], thus it could be argued that a selective reduction in CD8 vs. CD4 T cell accumulation via CNP’s reduction in gene expression mediated by IFN-γ is an early protective response by the endothelium. 

CNP exerts the vast majority of its effects via the GC-B receptor, and the generation of cGMP [33,34]. In our current study, not only did we confirm the presence of functional GC-B receptors in HUVEC, as described previously [44], but were able to mimic the dampening effects of CNP on IFN-γ responsiveness by using the cell permeable analogue, 8-Br-cGMP and with an additional cGMP analogue 8-CPT-cGMP, which is a more membrane permeant molecule. Interestingly, 8-CPT-cGMP only partially recapitulated the effects on MHC-I and ICAM-I seen in the presence of either CNP or 8-Br-cGMP. It is unclear as to why 8-CPT-cGMP failed to inhibit INF-y-stimulated ICAM-1 expression in HUVECs. However, previous studies suggest that the sub-cellular localisation of cGMP generation in cardiomyocytes and HUVEC can influence biological responsiveness [44,45]. Therefore, it is possible that CNP, 8-Br-cGMP and 8-CPT-cGMP treatments lead to spatially distinct increases in cGMP within HUVECs that may alter endothelial cell responsiveness.

HUVEC also express the NPR-C receptor, which has been strongly implicated in the role that CNP performs to maintain vascular homeostasis [46]. Even though NPR-C lacks intrinsic guanylyl cyclase and, therefore, does not directly enhance cGMP production, previous studies have shown that activation of NPR-C by natriuretic peptides may also stimulate nitric oxide production, leading to cGMP generation via soluble guanylyl cyclase [47]. Therefore, although our data implicate GC-B/cGMP signaling as a mechanism for the anti-inflammatory effects of CNP in HUVEC, it is perfectly conceivable that additional, NPR-C-mediated mechanisms may also contribute to these effects. 

We have used HUVEC as model human endothelial cells in this study because umbilical cords are a readily available, ethically uncontroversial source of human vascularized tissue. HUVEC have been used for more than 40 years and their responses to a range of stimuli including IFN-γ are very well characterized and have been shown to be comparable to adult human large artery endothelium [48]. 

In conclusion, CNP is able to downregulate IFN-γ-mediated gene expression in the endothelium, which could limit vascular inflammation by directing specific T cell subsets into developing atherosclerotic lesions, ultimately affecting atheroma progression. Further work is required to determine whether modulation of endogenously expressed endothelial CNP or addition of exogenous CNP is of potential therapeutic value to treat atherosclerosis in vivo.

## Supplementary Materials

The following are available online at http://www.mdpi.com/2079-6374/8/3/86/s1, Figure S1: ICAM-1 and MHC Class I expression in HUVEC after incubation with IFN-γ alone or in combinations with 8-CPT cGMP for 48 h.
