# Supplementary material for: C-Type Natriuretic Peptide (CNP) Inhibition of Interferon-γ-Mediated Gene Expression in Human Endothelial Cells In Vitro
Source: Biosensors (Basel). 2018 Sep 14;8(3):86. doi: 10.3390/bios8030086 (PMC6164118; doi:10.3390/bios8030086)
Supplement: Supplementary file 1 [file biosensors-08-00086-s001.pdf]

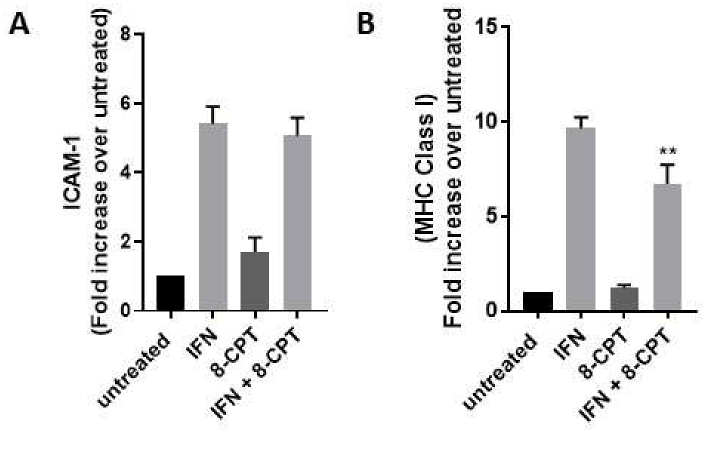

Supplementary Figure 1: ICAM-1 and MHC Class I expression in HUVEC after incubation with IFN-γ alone or in combinations with 8-CPT cGMP for 48 h. Mean Fluorescence Intensity (expressed as mean ± SEM fold increase over MFI of untreated cells), for (**A**) ICAM-1, (**B**) MHC Class I on untreated HUVEC or after 48 h treatment with IFN-γ alone or in combination with 8-CPT-cGMP; $n = 3$ HUVEC isolates (**$p < 0.01$, significantly different from IFN-γ alone).
